# Patients with endometriosis using positive coping strategies have less depression, stress and pelvic pain

**DOI:** 10.1590/S1679-45082017AO3911

**Published:** 2017

**Authors:** Lilian Donatti, Denise Gimenez Ramos, Marina de Paula Andres, Leigh Jonathan Passman, Sérgio Podgaec

**Affiliations:** 1 Pontifícia Universidade Católica de São Paulo, São Paulo, SP, Brazil.; 2 Hospital das Clínicas, Faculdade de Medicina, Universidade de São Paulo, São Paulo, SP, Brazil.; 3 University of California, Los Angeles, CA, United States.; 4 Instituto Israelita de Ensino e Pesquisa Albert Einstein, Hospital Israelita Albert Einstein, São Paulo, SP, Brazil.

**Keywords:** Endometriosis, Adaptation, psychological, Depression, Stress, psychological, Pelvic pain

## Abstract

**Objective:**

To determine the correlations between coping strategies, depression, stress levels and pain perception in patients with endometriosis.

**Methods:**

This prospective and exploratory study included 171 women undergoing treatment for endometriosis between April and August 2014. The questionnaires used were Brief COPE, Beck Depression Inventory, Lipp’s Stress Symptom Inventory for Adults and Visual Analogue Scale. Clinical data were collected from electronic medical records.

**Results:**

Patients with endometriosis who used positive coping strategies had better adaptation to stress (p<0.004) and less depression (p<0.004). The presence and intensity of depression, stress and acyclic pelvic pain were directly associated (p<0.05). The intensity of dysmenorrhea was associated with the degree of depression (p<0.001), whereas acyclic pelvic pain was associated with the degree of depression (p<0.001), stress level (p<0.001) and stress type (p<0.001).

**Conclusion:**

We found a positive association between coping, depression levels, type and levels of stress and pain intensity in patients with endometriosis. The use of maladaptive coping strategies focused on emotion is correlated with increase in depression and stress.

## RESUMO

**Objetivo:**

Observar a correlação entre estratégias de enfrentamento, depressão, níveis de estresse e percepção de dor em pacientes com endometriose.

**Métodos:**

Estudo prospectivo e exploratório, que incluiu 171 mulheres em tratamento por endometriose entre abril e agosto de 2014. Foram utilizadas as escalas: COPE Breve, Inventário de Depressão de Beck, Inventário de Sintomas de Stress para Adultos de Lipp e a Escala Visual Analógica. Os dados clínicos foram coletados do prontuário eletrônico.

**Resultados:**

Pacientes com endometriose que utilizaram estratégias positivas de enfrentamento apresentaram melhor adaptação ao estresse (p<0,004) e menos depressão (p<0,004). A presença e a intensidade da depressão, do estresse e da dor pélvica estiveram diretamente associadas (p<0,05). A intensidade da dismenorreia foi associada com o grau de depressão (p<0,001), enquanto a intensidade da dor pélvica acíclica esteve associada com o grau de depressão (p<0,001), nível de estresse (p<0,001) e tipo de estresse (p<0,001).

**Conclusão:**

Houve associação positiva entre *coping*, níveis de depressão, tipo e níveis de estresse e intensidade da dor nas pacientes com endometriose. A utilização de estratégias de *coping* desadaptativa focada na emoção está correlacionada com o aumento da depressão e do estresse.

## INTRODUCTION

Endometriosis is a chronic inflammatory disease featured by presence of endometrial cells outside the uterine cavity. The precisely prevalence of endometriosis is unknown, it affects 2 to 10% of women at reproductive age and can achieve approximately 50% of women with pelvic pain^1,2^. The diagnosis and experience with the disease can involve a number of spheres in a woman’s life, such as, physical,^3-5^ emotional,^6,7^ marital, sexual,^8,9^ professional^10,11^ and psychological.^3-5,12-14^

To discover the disease is undoubtedly an important event in women’s emotional life, and the fast and precise diagnosis is fundamental in order to ameliorate suffering and distress of waiting for answers and treatment plans. For this reason, the clinical evaluation, followed by specific image exams such as transvaginal ultrasound with intestinal preparation, enables experts to define an adequate therapeutic strategy.^15^

A number of studies have approached the subject of endometriosis, but they observe a gap in the psychological aspect of the disease.^1^ Treatment of emotional symptoms along with physical symptoms might bring great benefits and it may constitute the most powerful therapeutic result.^12^ Based on psychosomatic theory, it is impossible to differentiate influence that mind causes in the body and vice-versa, this is an unique and indissoluble proportion.^12^ Therefore, a tendency exists to consider psychosomatic all diseases as they involve the continuous inter-relation between body and mind in its origin, development and healing.

Studies approaching psychological perspective of endometriosis deal with issues such as patients’ poor quality of life,^10,14,16-18^ including harms in interpersonal and affective relationships;^6,16^ difficulties in sexuality;^9,19^professional losses;^10,11^depression and anxiety;^3,7,20^ suffering facing the recognition of healing difficulties;^21^ways for facing the disease;^4^constant presence of pain and stress;^4,5,22,23^ importance of therapy and group support;^3,5,12-14^and other complementary therapies in which the objetive is to reduce pain and anxiety, such as acunputure and relaxing techniques,^24,25^ exercises^26^ and changes in eating habits.^1^

To understand life of patients with endometriosis and comprehend their coping strategies seem to be initial of an important pathway to develop a more comprehensive treatment and provide resources for future interventions.

## OBJECTIVE

To observe correlation between coping strategies, depression, levels of stress and perception of pain in patients with endometriosis.

## METHODS

This was a prospective and exploratory study with quantitative methodological approach. The study was approved by Ethical Committee of *Pontifícia Universidade Católica de São Paulo*, statement number 701.681/2014, CAAE number 26209813.7.0000.5482, and by Ethical Commitee of *Hospital das Clínicas da Faculdade de Medicina da Universidade de São Paulo* (USP), statement number 741.946/2014, CAAE number 26209813.7.3001.0068.

Data were collected between April and August 2014 in an outpatient unit of endometriosis sector of the Department of Gynecology and Obstetrics of the *Hospital das Clínicas*. We included women aged between 18 and 45 years old, who agreed to participate in the study and signed the consent form, with suggestive imaging for endometriosis injury by transvaginal ultrasound with intestinal preparation or pelvic magnetic resonance performed at *Hospital das Clínicas* and conducted by a specialized team. Exclusion criteria were mistakes to complete questionnaires and medical records with missing data.

During the period of the study, 523 patients were assisted in the endometriosis center at outpatient unit. Of these, we selected 191 who responded questionnaires. The same researcher applied surveys, and after application of exclusion criteria, 171 patients were included in the study.

Epidemiological and clinical data were collected from electronic medical records. Typical symptoms related to presence of the disease (dysmenorrhea, chronic pelvic pain, deep dyspareunia, cyclical intestinal change, cyclical urinary change, and infertility) were classified from 0 to 10 using the Visual Analogue Scale (VAS).^27^ Pain intensity was classified as mild (1 to 3 points), moderate (4 to 7 points), or severe (8 to 10 points). Endometrosis type was classified as superficial (when identified only injuries with less infiltration than 5mm of peritoneum), ovarian (in presence of cystic endometriosis and lack of deep injury) or deep (injuries win greater infiltration than 5mm).^28^

After, we applied the Brief Coping Orientation to Problems Experienced (COPE),^6^ the Beck Depression Inventory (BDI)^29^, and the Lipp’s Stress Symptom Inventory for Adults (LISS).^30^The brief COPE^6^ is a questionnaire with 28 multiple choice questions, measuring the frequency in which a patient presents or not specific described behaviors, and which the result ends-up presenting their coping strategy that can be “focused on the problem” (which is considered positive); “adapted focused on emotion” (also positive, but emotions can affect the action) or “unadaptive focused on the emotion” (considering negative, because there is scape or avoidance behavior from the problems). The Beck Depression Inventory^29^ is a self-assessment instrument including 21 items concerning symptoms and aptitudes that identify the presence or absence of depression. The result is presented in score that can be 0 to 13 (without depression or minimal depression), from 14 to 19 (mild depression), from 20 to 28 (moderate depression) and 29 to 63 (severe depression). The LISS^30^ is an instrument that can be divided into three blocks and evaluate the group symptoms experienced within the last 24 hours, in the last week and in the last month. The result classifies the stress into phases designed as alerting, resistance, almost exhaustion and exhaustion, and the quality of stress, which can be physical, psychological, or physical- psychological.

### Statistical analysis

Data collected were analyzed using the Statistical Package for the Social Sciences (SPSS), version 20.0. Each applied scale was corrected according to its respective manuals, and general results obtained were expressed in categories indicated by authors. Qualitative variables were compared using the χ^2^ test. Level of significance adopted was 0.05 to all variables.

## RESULTS

Patients mean age was 35.9±5.6 years, and most of them were married (44.4%) and had completed high-school (48.2%). The affecting of endometriosis was superficial in 2.9%, ovarian 18.2% or deep in 78.8% of cases. Among symptoms presented, 42.7% had infertility, 87.1% dysmenorrhea and 90.6% chronic pelvic pain, and this last was classified as severe (VAS >7) in 39.8%.

In relation to emotional aspect, most of the patients never conducted psychological (93.6%) or psychiatric (87.1%) treatment. Among patients included, 66.3% (n=108) used positive coping strategy as “focused on the problem”, 26.9% (n=44) “adaptative coping focused in the emotion” and 6.7% (n=11) unadaptive focused in the emotion”. Most of the women (53.8%) had some degree of depression, which was minimal/mild in 68.4%, moderate/severe in 31.6%. Similarly, the majority of patients had stress (62.5%) and 41.5% had psychological stress.

In order to verify how coping strategies influence depression, in stress and pain because of endometriosis, we performed crossings among studied variables in order to observe the existence of association between them.

Analysing table 1, we understand that depression in severe level influenced action and decision making in coping strategies of daily life situations. More stressed patients were those who used more than one unadaptive strategy in coping situations whereas less stress solved the issues focusing in the problem (p<0.004). In relation to coping and depression variables, we observed that the more depressive patient had greater unadaptive strategy to deal with daily life problems (p<0.004).

**Table 1:** Coping association with stress and depression level in women with endometriosis

Characteristics	Brief COPE			p value

	Focused on the problem (n=108)	Adaptive focused on the emotion (n=44)	Unadaptive focused on the emotion (n=11)	
Stress level^*^				
No stress (n=62)	43 (69.4)	18 (29.0)	1 (1.6)	<0.004^†^
Alert/resistence (n=83)	56 (67.5)	22 (26.5)	5 (6.0)	
Exhaustion/almost exhaustion (n=18)	9 (50.0)	4 (22.2)	5 (27.8)	
Depression level^‡^				
Minimal/mild (n=112)	83 (76.9)	28 (63.6)	1 (9.1)	<0.004^§^
Moderate/severe (n=51)	25 (23.1)	16 (36.4)	10 (90.9)	

* Lipp’s Stress Symptom Inventory for Adults (LISS). ^†^ χ^2^ test: 15,364; ^‡^ Beck depression inventory; ^§^ χ^2^ test: 22,043.

In relation to depression and stress, there an association between level of stress and depression degree (p<0.001; table 2). Psychological stress had prevalence of 66.7% in those who had moderate/severe depression and 29.9% of those who had minimal/mild depression, which showed a significant association between type of stress with level of depression (p<0.0001).

**Table 2:** Association between type/level of stress and levels of depression in women with endometriosis

Stress*	Level of depression^†^		Total (n=171)	p value

	Minimal/mild (n=117)	Moderate/severe (n=54)		
Type				
No stress	59 (50.4)	5 (9.2)	64 (37.4)	<0.0001^‡^
Physical	19 (16.2)	5 (9.2)	24 (14.0)	
Psychological	35 (29.9)	36 (66.8)	71 (41.5)	
Physical and Psychological	4 (3.4)	8 (14.8)	12 (7.0)	
Level				
No stress	59 (50.4)	5 (9.2)	64 (37.4)	<0.001^§^
Alert/resistence	53 (45.2)	36 (66.8)	89 (52.0)	
Exhaustion/almost exhaustion	5 (4.2)	13 (24.0)	18 (10.5)	

^*^ Lipp’s Stress Symptom Inventory for Adults (LISS). ^†^ Beck depression inventory; ^‡^ χ^2^ test 33,734; ^§^ χ^2^ test: 36,871.

The intensity of dysmenorrhea pain was directly related with presence and severity of depression. In 91.7% of patients with severe pain (VAS>7) we observed moderate or severe depression (p<0.001; table 3).

**Table 3:** Association between level of dysmenorrhea and depression in women with endometriosis

Dysmenorrhea (VAS)*	Levels of depression^†^			p value^‡^

	Minimal/mild (n=104)	Moderate/severe (n=48)	Total (n=152)	
No	3 (2.9)	0 (0)	3 (2.0)	<0.001
Mild/moderate	27 (26.0)	4 (8.3)	31 (20.4)	
Severe	74 (71.2)	44 (91.7)	118 (77.6)	

* VAS: 1-3 points for mild pain; 4-7 points for moderate pain; 8-10 points for severe pain; ^†^ Beck depression inventory; ^‡^ χ^2^ test: 24,178. VAS: Visual Analogue Scale.

Presence of chronic pelvic pain was also associated with phases, stress types and depression (Table 4). Only 20.3% of women who had chronic pelvic pain in any intensity presented stress, however, when pain was classified as severe, stress prevalence was 78.2%. In addition, we observed that higher stress severity showed higher score in analogical pain scale (p<0.001)*.*

**Table 4:** Association between chronic pelvic pain, level of depression, and type/level of stress in women with endometriosis

	n (%)	Chronic pelvic pain (VAS)^*^			p value

		No pain (n=16)	Mild/moderate (n=87)	Severe (n=68)	
Stress level^†^					
No Stress	64 (37.4)	13 (20.3)	34 (53.1)	17 (26.6)	
Alert/resistence	89 (52.0)	3 (3.4)	49 (55.1)	37 (41.6)	<0.001^‡^
Exhaustion/almost exhaustion	18 (10.5)	0 (0)	4 (22.2)	14 (77.8)	
Type of stress^†^					
No stress	64 (37.4)	13 (20.3)	34 (53.1)	17 (26.6)	
Physical	24 (14.0)	1 (4.2)	11 (45.8)	12 (50.0)	<0.001^§^
Psychological	71 (41.5)	2 (2.8)	32 (45.1)	37 (52.1)	
Physical/psychological	12 (7.0)	0 (0)	10 (83.3)	2 (16.7)	
Depression^¶^					
Minimal/mild	117 (68.4)	16 (13.7)	66 (56.4)	35 (29.9)	<0.001||
Moderate/Severe	54 (31.6)	0 (0)	21 (38.9)	33 (61.1)	

* VAS: 1-3 points for mild pain; 4-7 points for moderate pain; 8-10 points for severe pain; ^†^ Lipp’s Stress Symptom Inventory for Adults (LISS), ^‡^ χ^2^ test: 26,996, ^§^ χ^2^ test: 24,178, ^¶^ Beck depression inventory; || χ^2^ test: 18,656. VAS: Visual Analogue Scale.

All patients with endometriosis and severe depression had clinical complaint of pelvic pain and, the higher the depression picture the greater the perception of chronic pain (p<0.001).

## DISCUSSION

To our best knowledge, this is the first study correlating coping strategies, depression, stress and pelvic pain in endometriosis. Mello^31^ studied coping strategy in 30 patients with endometriosis and found that 60% of them used assertive coping strategies. In our data, we found 66.3% of women using assertive coping strategies focused on problem. Indexes are quite close and show similariteis in behavior of these patients.

Annual prevalence of depression in general population ranges from 3 to 11%.^32^ In patients who were admitted because of any physicial disease varied three times more in women than in men.^32^ In studies that related endometriosis and depression, only two studies showed similar indexes in patients with endometriosis and pelvice pain. One study^20^ found frequency of 86% of depression, and 52% was moderate/severe, the second^7^ observed a prevalence of 86.5% of depression, and 63.5% of moderate/severe depression. In our study, prevalence of patients with depression was 53.8%, and 31.6% of moderate and severe depression. This difference can be related to the fact that these patients were assisted in tertiary center for treament of endometriosis and aproximately 80% of them had deep injuries that is the most aggressive type of the disease.

We did not find any study that used the Lipp’s questionnarie^30^to measure stress in patients with endometriosis, but there are studies, using other methods. In addition, we also observed high stress indices among these women.^14,22,23^ In this study, we observed 62.2% of women with stress, and psychologic stress was predominant in 41.5% of cases. A number of studies approach the binomy of pain symptoms with endometriosis. Martin et al.^4^ studied 115 women with endometriosis and chronic pelvic pain, correlating the variables pain, quality of life, and psychosocial factors including catastrophic coping style. In general, 43.5% of patients pointed out moderate to severe level in “catastrophizing”. The catastrophizing degree was a significant predictor of pain during the follow-up: among women who presented severe catastrophizing in initial evaluation, the pain degree was higher in the beginning and in the ending of the study compared to moderate and mild degree of catastrophizing. In addition, only those with mild (p<0.001) and moderate (p<0.001) catastrophizing degree had reduction of painful symptom within 1 year.

Results suggest that bio psychosocial factors, especially in terms of catastrophyzing, could be associated with untreated pain. Authors suggest that in literature on pain there is a debate if catastrophyzing is the cause or consequence of chronic pain.^4^ These authors concluded that, so far, there are no longitudinal studies analyzing if catastrophyzing is modified by a persistent pain status.

Women with severe pelvic pain sometimes have comorbidities such as muscular pain of pelvic floor and poor quality of life. Martin et al. highlighted that the small size of the sample used in their, it enbled them to study group and independent contributions of other known psychological variables (*e.g.*, history of trauma and abuse), and they also indicated that because psychological factors had a significant influence over the pain report, further large studies are urgently needed to examine contributions on neurobiological and psychological domains in the origin and treatment of persistent pain status in women with endometriosis.^4^

In our analysis, 87.1% of patients had dysmenorrhea and 90.6% chronic pelvic pain. We observed an association between pain intensity and depression: among those with moderate to severe depression, 91.7% had severe dysmenorrhea (p<0.001) and 61.1% severe chronic pelvic pain (p<0.001). Intensity of painful symptoms was also associated with the presence and intensity of stress. In addition, depression and stress were associated among them, and psychological type of stress was prevalence in this relationship.

Similarly, Lorençatto et al*.* ^3^studied 128 women with endometriosis and showed that 76.6% of them had pain associated with depression. In the end of intervention, after 10 weekly meetings including physiotherapy exercises and psychological activites based in the behavioral cognitive approach, they identified a significant reduction of pain and depression score.

In our study, we observed that patients who used positive coping strategies to face the disease had less stress and depression. This fact may occur because depression and stress in more severe levels tend to cause higher emotional vulnerability. This result is of clinical relevance because it suggests the need of implement an intervention to assist these patients to develop assertive coping strategies that can help to improve depression and stress, and in this way, to improve treatment offered for these women.

This study had some limitations: the institutions in which the research was done is a tertiary referral center for treatment of endometriosis, in such a way, that most of patients included presented severe cases of the disease, and it could be important factor of biased in the obtained result. The fact of inexistence of similar studies in literature limits comparison and extrapolation of results obtained. Finally, the size of the sample and design of the study are also important factors for of not including a control group for comparison of variables.

Our study confirmed the importance of treating endometriosis in terms of a psychosomatic point of view, because its symptoms affect the patient in physical and emotional levels as well, as we proved by the association among studied variables. The endometriosis is a disease of multiple facets and influence life of patients in a number of levels, therefore it should be treated in this same dimension.

## CONCLUSION

We observed an association between coping strategy, depression, type and levels of stress, pain intensity in patients with endometriosis. Therefore, clinical treatment of the disease should include the psychic approach of symptom with intervention based in coping strategies theory and reduction of stress, thus contributing to reduce depression and symptoms related to endometriosis.
